# Infection with SARS-CoV-2 Omicron Variant 24 Days after Non-Omicron Infection, Pennsylvania, USA

**DOI:** 10.3201/eid2809.220539

**Published:** 2022-09

**Authors:** Arlene G. Seid, Tigist Yirko, Sameera Sayeed, Nottasorn Plipat

**Affiliations:** Pennsylvania Department of Health, Bureau of Epidemiology, Harrisburg, Pennsylvania, USA (A.G. Seid, N. Plipat);; Pennsylvania Department of Health, Bureau of Epidemiology, Lancaster, Pennsylvania, USA (T. Yirko);; Pennsylvania Department of Health, Bureau of Laboratories, Exton, Pennsylvania, USA (S. Sayeed)

**Keywords:** COVID-19, coronavirus disease, SARS-CoV-2, severe acute respiratory syndrome coronavirus 2, viruses, respiratory infections, zoonoses, vaccine-preventable diseases, Pennsylvania, United States

## Abstract

A 42-year-old man, with up-to-date COVID-19 vaccination, experienced symptomatic SARS-CoV-2 infection in December 2021. Mutation tests suggested a non-Omicron variant. After his recovery, and 24 days after the first positive SARS-CoV-2 test, he had onset of symptomatic infection with the BA.1.1 (Omicron) variant, which was confirmed by whole-genome sequencing.

Repeated positive findings for SARS-CoV-2 infection within 90 days pose diagnostic challenges for public health professionals. Such results imply persistent viral shedding, reinfection, or coinfection, and each determination requires a different isolation and quarantine approach. When genetic sequencing resources are limited, healthcare professionals must base risk assessment decisions on such criteria as exposure history and community transmission levels. We describe a vaccinated healthcare worker who had positive SARS-CoV-2 tests 24 days apart. Each positive test was associated with a separate symptomatic illness.

On December 20, 2021, a 42-year-old otherwise healthy man, employed in a nursing home, had onset of nausea and emesis. He was up to date with COVID-19 vaccinations, having received the 2 initial doses of the Pfizer-BioNTech vaccine (https://www.pfizer.com), as well as a booster dose on October 11, 2021. He tested positive for SARS-CoV-2 by real-time reverse transcription PCR (RT-PCR) using Taqman assays (Thermo Fisher Scientific, https://www.thermofisher.com). The PCR test detected nucleocapsid 1 protein (cycle threshold [Ct] 33), nucleocapsid 2 protein (Ct 28), and spike protein (Ct 33) genes and did not detect the open reading frame 1ab gene. Further mutation tests by TaqMan Mutation Detection Assays (Thermo Fisher Scientific) showed the absence of delH69V70, suggesting the patient’s infection was probably not caused by the Omicron BA.1 variant. The patient recovered within 1 week.

On January 12, 2022, the patient had new onset of fever, chills, myalgia, and cough. Four of his 6 household members were also sick and received positive results after administration of SARS-CoV-2 at-home antigen tests (Figure). The patient was tested at an urgent care clinic. The Quidel QuickVue SARS antigen test (Quidel, https://www.quidel.com) showed a positive result, and the BD Veritor influenza A/B antigen test (Thermo Fisher Scientific) showed a negative result. Negative findings from a multiplex RT-PCR for respiratory pathogens eliminated consideration of alternative diagnoses. The patient’s specimen was sent to the Pennsylvania Department of Health Bureau of Laboratories (BOL) and tested by the CDC Influenza SARS-CoV-2 (FluSC2) Multiplex RT-PCR Assay. The test result was negative for Influenza A and B, but positive for SARS-CoV-2 (Ct 19). The whole-genome sequencing (Illumina, https://www.illumina.com) yielded Omicron variant BA.1.1. The patient tested negative by RT-PCR 1 week later.

**Figure:**
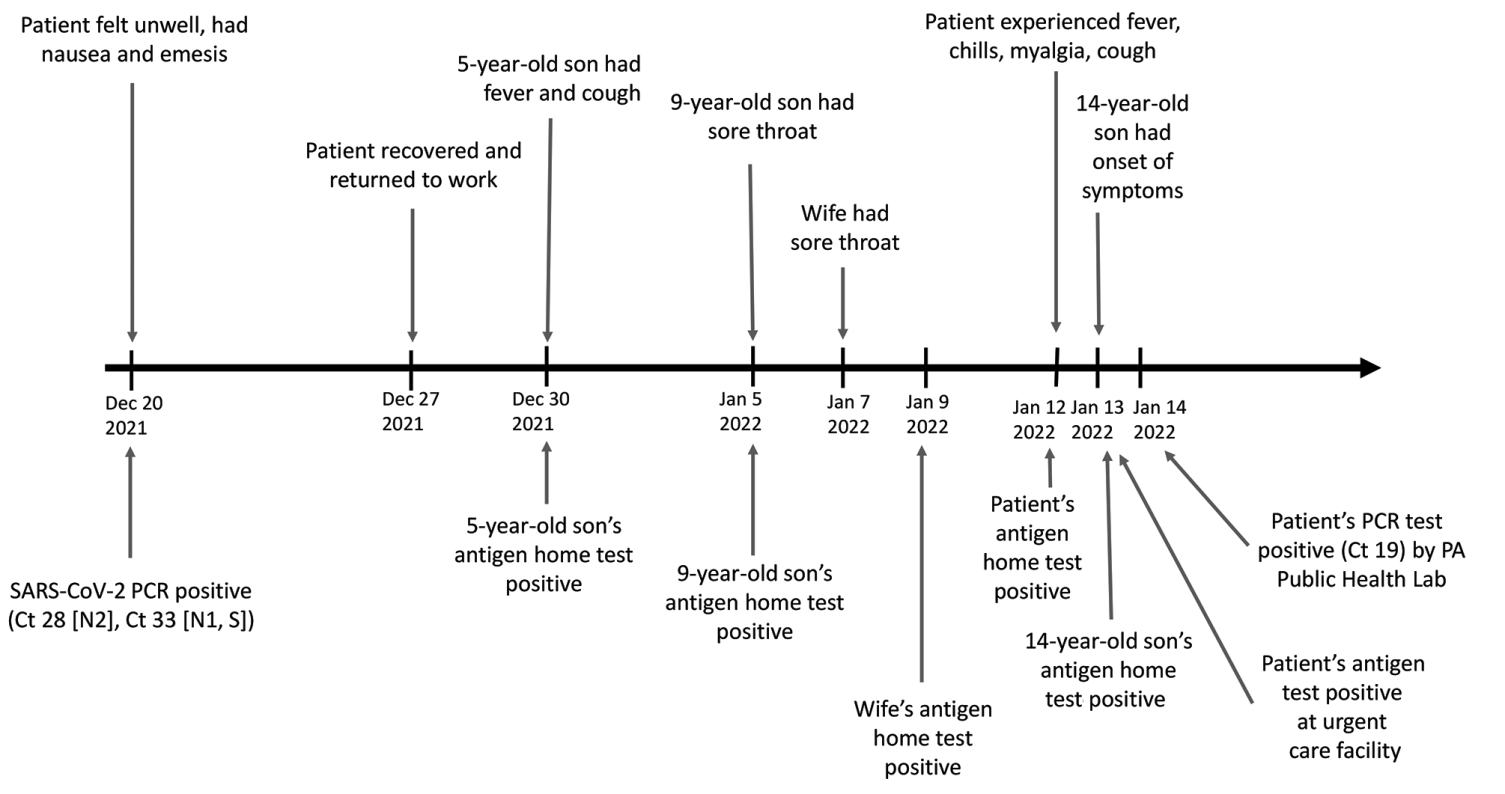
Timeline of a vaccinated healthcare worker who had positive viral tests for SARS-CoV-2 infection 24 days apart (December 20, 2021, and January 12, 2022), Pennsylvania, USA. Image shows symptoms and test results for the patient and household members. The patient and his wife were up to date with Pfizer-BioNTech (https://www.pfizer.com) SARS-CoV-2 vaccines (2 doses of primary series and 1 booster dose). Both eligible children (9-year-old and 14-year-old sons) were fully vaccinated against SARS-CoV-2. Ct, cycle threshold; N1, nucleocapsid 1 protein; N2, nucleocapsid 2 protein; PA, Pennsylvania; S, spike protein.

Reinfection with a different virus variant is the most likely explanation for the positive antigen and PCR tests 24 days after this patient’s initial SARS-CoV-2 infection diagnosis. We base this assumption on 3 facts: the symptomatic illnesses were separated by a full, albeit brief, recovery period; tests uncovered 2 genotypically distinct variants; and household exposure presented a likely route of transmission for the second infection during an Omicron surge. 

Studies have described co-infections with 2 SARS-CoV-2 variants; however, those co-infections were noted either as contributors to a singular illness or as co-detected events in the same samples (*1*,*2*). Although persistent positive test results may follow an asymptomatic period, the onset of new symptoms and subsequent confirmation of a different variant by whole-genome sequencing makes that explanation unlikely for the patient we studied.

The frequency of coronavirus reinfection has been shown to depend on many variables: the studied population, the SARS-CoV-2 variants, time and place, and the defined duration between the initial and subsequent infections. The interval between infections of the same seasonal coronavirus could be <12 months (*3*). For SARS-CoV-2, the interval between reported infections of genetically distinct variants has ranged from 23 to >90 days (*4*).

Although this case appears to lend support to prior studies demonstrating the capacity of the Omicron variant to evade immunity, our findings also suggest that a fully protective humoral and cell-mediated immunity might take longer than 24 days to develop (*5*,*6*). Antibodies to SARS-CoV-2 infection may be present as early as 10 days postinfection, but the presence of antibodies alone is an incomplete predictor of protection (*7*). Cross-reactive immunity after COVID-19 illness and SARS-CoV-2 vaccination has been shown to confer broad protection against heterologous coronaviruses. This protection, however, might be variable depending on variants (*8*). When compared with ancestor and other variants, the Omicron variant has been shown to demonstrate reduced neutralization (*9*). Convalescent serum from infected patients largely did not neutralize the Omicron variant; conversely, serum from infected patients who were subsequently vaccinated and from patients who were vaccinated and had breakthrough infections did neutralize the Omicron variant, but to a lesser degree than for the Delta variant (*9*). In the patient we describe, immune response from 3 mRNA vaccines and COVID-19 infection did not prevent reinfection.

As documented in another study, household secondary attack rate by Omicron is higher (25%) than for the Delta variant (11%), even among booster-vaccinated persons (F.P. Lyngse et al., unpub. data, https://doi.org/10.1101/2021.12.27.21268278). In the patient we describe, it is more likely that household exposure led to the second infection. Still, given the short interval (24 days) between the 2 infections and the unavailable genetic sequencing data, we cannot rule out that this patient’s initial infection might have been the source of the subsequent infections among members of the household. Full assessment of the clinical context, individual risk exposure, and community transmission level is essential in determining diagnosis and appropriate health intervention in patients who test positive again shortly after an initial positive viral test for SARS-CoV-2 infection. 
